# Prevalence and Risk Factors for Minor Hallucinations in Patients with Parkinson's Disease

**DOI:** 10.1155/2021/3469706

**Published:** 2021-10-04

**Authors:** Min Zhong, Ruxin Gu, Sha Zhu, Yu Bai, Zhuang Wu, Xu Jiang, Bo Shen, Jun Zhu, Yang Pan, Jun Yan, Li Zhang

**Affiliations:** ^1^Department of Geriatric Neurology, Affiliated Brain Hospital of Nanjing Medical University, Nanjing, China; ^2^Department of Biological Sciences, University of Toronto Scarborough, Canada; ^3^Institute of Neuropsychiatric Diseases, Brain Hospital Affiliated to Nanjing Medical University, Nanjing, China

## Abstract

**Purpose:**

As the most frequent and earliest type of psychotic phenomenon in Parkinson's disease (PD), minor hallucination (MH) can occur before the onset of motor symptoms. This sensation may be an early predictor of severe psychotic and cognitive states and is often overlooked in clinics. This study was aimed at providing a comprehensive and in-depth understanding of MHs. *Patients and Methods*. Demographic information was obtained from 262 patients with PD, and a series of clinical assessment questionnaires were provided. According to the result of the Movement Disorders Society Unified Parkinson's Disease Rating Scale Part I, the patients were classified into the MH and nonhallucination (NH) groups.

**Results:**

MHs were the most common psychotic symptom with 38.9% prevalence. The most frequent MH was visual illusion, especially object misidentification. Three minor phenomena were somewhat consistent in terms of external factors, temporal factors, and content. Disease duration, daily levodopa equivalent dose, and percentage of levodopa and dopamine-receptor agonist use were remarkably greater in the MH group than in the NH group. After covariate control, the MH group had worse life quality and more severe nonmotor symptoms, including poor sleep quality and rapid eye movement sleep behavior disorder (RBD), than the NH group. The binary logistic regression model showed that RBD, sleep quality, and health-related life quality were associated with MHs.

**Conclusion:**

A high prevalence of MHs was observed in patients with PD. Further studies are needed to confirm and expand the identified clinical factors related to MH, which have potential prognostic and therapeutic implication.

## 1. Introduction

Parkinson's disease (PD) is the second largest degenerative disease that has affected a large number of people worldwide [1]. As one of the most detrimental nonmotor symptoms, PD associated psychosis (PDP) affects up to 75% of patients throughout the disease course [2]. This prevalence is underestimated because the psychotic symptoms are not fully considered as a spectrum that includes minor hallucinations (MHs), major hallucinations, and delusions.

In 2000, Fénelon et al. systematically described the minor hallucinatory phenomena that consequently prompted attention to MH [3]. Seven years later, MHs were included in the diagnostic criteria for PDP by the National Institute of Neurological Disorders and Stroke and National Institute of Mental Health group [4]. Nishio et al. further defined and concluded visual illusions [5]. Three types of phenomena are now grouped under the title of MHs: presence hallucinations, passage hallucinations, and visual illusions. Presence hallucination refers to feeling a presence of someone nearby when the patients are focusing their attention on something. Passage hallucination occurs when the patients catch a glimpse of a passing fuzzy shadow that is often reported as a person or an animal. Visual illusions include object misidentification illusions (seeing something as another object with a similar shape), pareidolias (seeing human faces or others from complex patterns), and kineptosia (seeing still life as moving) [6].

Major hallucinations include well-structured visual hallucinations (VHs) and hallucinations in other senses, such as auditory, olfactory, gustatory, and tactile [7]. Especially well-structured VHs, these phenomena have been heavily researched and thoroughly described. Well-structured VHs have a 50% lifetime prevalence [8] and more than 80% cumulative prevalence [9, 10]. Progressive and recurrent VH is often associated with a likelihood of poor life quality, increased patient hospitalization, and nursing home admission [11, 12]. In addition, this is a risk factor for dementia and is associated with the high mortality rate [13]. At present, multiple models including the Perception and Attention Deficit model [14] have been proposed to explain the mechanisms of well-structured VHs in PD to some extent.

Although MHs are now recognized to be closely associated with well-structured VHs [15], it has not been studied as thoroughly as well-structured VHs. MHs deserve widespread attention because it is the most frequent and earliest type of psychotic phenomenon in PD and occurs even before the onset of motor symptoms [16]. MHs might also be an early predictor of a severe psychotic and cognitive state [6]. Hence, this work was aimed at providing a comprehensive and in-depth understanding of MHs in the Chinese population.

## 2. Material and Methods

### 2.1. Patients and Study Design

Patients with PD (*n* = 262) were consecutively recruited from the Affiliated Brain Hospital of Nanjing Medical University between May 2019 and January 2021. At least two experienced movement-disorder specialists participated in the diagnosis of PD. Participants were eligible for inclusion if they met the PD United Kingdom Brain Bank criteria [17] and could accomplish a series of questionnaires completely and accurately. The one-year rule was strictly enforced to distinguish dementia with Lewy bodies (DLB) and PD with dementia [18]. Subjects were excluded if they had a history of major psychiatric diseases or use of any antipsychotic medication. All patients had an MRI scan prior to this study to exclude the possibility of abnormalities known to impair mental status other than PD. Other exclusion criteria were abnormal vision or corrected vision.

The subjects were categorized into eight strata, namely, no hallucination, isolated minor hallucination, isolated major hallucination, isolated delusion, and the combination of these psychiatric symptoms, on the basis of their response to the Hallucinations and Psychosis item of the Movement Disorder Society-sponsored revision of the Unified Parkinson's Disease Rating Scale (MDS-UPDRS) part 1:0 = no hallucinations, 1 = minor hallucinations, 2 = formed hallucinations with insight, 3 = formed hallucinations without insight, and 4 = delusions [19]. Patients with a score of 0 in this item were included in the PD-NH group (*n* = 141), and those with a score of 1 and fulfilled the condition in which MHs occurred steadily over the last two months prior to this study were considered to have isolated MHs and were enrolled in the PD-MH group (*n* = 74).

All participants signed informed written consent and provided clinical data. This study was approved by the local Ethics Committee of the Brain Hospital Affiliated to Nanjing Medical University and was conducted in accordance with the principles outlined in the Declaration of Helsinki.

### 2.2. Clinical Outcomes

All PD patients were interviewed using a standard questionnaire to collect basic information, including age, gender, marriage, education level, body mass index (BMI), allergies, history of smoking and drinking, history of drinking tea and coffee, daily exercise, hypertension, diabetes, lacunar infarction, family history of PD, predominance of motor symptoms, age of PD onset, disease duration, and use of antiparkinsonian medicine. Daily levodopa equivalent dose (LEDD) was also calculated [20].

Motor function was assessed using the Unified Parkinson's Disease Rating Scale motor subscales (UPDRS III) and the Hoehn and Yahr (H-Y) scale. UPDRS III was tested twice; the first assessment was performed after antiparkinsonian medication was stopped for at least 24 h (controlled release agent discontinued for at least 72 hours), and the patients fasted overnight (OFF state); the second was conducted 1 hour after the patients were administered with levodopa at 1.5 times their regular morning dose (ON state) [21]. Nonmotor symptoms were measured using the Non-Motor Symptoms Questionnaire (NMS-Quest) [22]. Global cognitive function was evaluated using the Montreal Cognitive Assessment (MoCA) [23]. The Parkinson's Disease Sleep Scale (PDSS) was used to measure sleep quality [24] and the Rapid Eye Movement Sleep Behavior Disorder Screening Questionnaire (RBDSQ) to further screen for RBD [25]. All the patients finished the Hamilton Anxiety Rating Scale (HAMA) and the Hamilton Rating Scale for Depression (HAMD) for the evaluation of the severity of anxiety and depression. Health-related quality of life was measured using the Parkinson's Disease Questionnaire-39 (PDQ39) [26].

The characteristics of MHs were assessed in more detail in the PD-MH group by using a questionnaire comprising 12 items. Based on our previous questionnaire [27], this edition did some adaption according to the definition of MHs and covered the nature and properties of MHs as much as possible.

### 2.3. Statistical Analysis

All continuous variables were presented as mean ± standard deviation (SD). After data normality was analyzed using the Kolmogorov–Smirnov test, Student's *t*-test and Mann–Whitney *U* test were conducted to examine differences between the PD-MH and PD-NH groups. All categorical variables were shown as percentages (%), and Chi square test, Pearson test, and Fisher's exact test were performed. Mann–Whitney *U* test was used for grade data, such as education level. The scores of nonmotor symptoms were recompared using one-way analyses of covariance (ANCOVA) after the adjustment for confounding factors, including disease duration and LEDD. Logistic regression was conducted to evaluate the related factors of MHs. Univariate regression analysis was first performed to screen statistically significant variables, and all meaningful variables were then submitted for multivariate regression analysis using an enter logistic regression. Two-sided *P* < 0.05 was considered statistically significant. SPSS 23.0 (IBM Corporation, New York, USA) was used for data analysis.

## 3. Results

### 3.1. Prevalence and Characteristics of MHs

**Figure 1:**
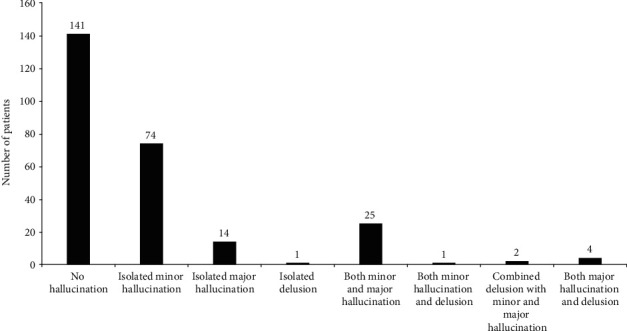
Prevalence of psychotic symptoms in the cohort. Of the 262 patients with PD recruited in our study, 102 (38.9%) experienced MHs. This included 74 patients (28.2%) with isolated MHs and 28 patients (10.7%) with combined MHs. Fourteen (5.3%) and one (0.4%) patients experienced isolated major hallucinations and delusions, respectively. Among the population, 32 (12.2%) had more than one kind of psychiatric symptoms. The remaining 141 (53.8%) reported having no hallucinations or delusion.

Among the 262 patients with PD recruited in our study, 102 (38.9%) experienced MHs, including 74 patients (28.2%) with isolated MHs and 28 patients (10.7%) with combined MHs. Isolated major hallucinations and delusions were presented in 14 (5.3%) and 1 (0.4%) patients, respectively. Up to 32 (12.2%) patients had more than one kind of psychiatric symptoms. The remaining 141 (53.8%) reported having no hallucinations or delusion. The result is shown in Figure 1.  Table 1 shows the characteristics of MHs. A total of 122 MHs were detected in 74 patients with isolated MHs. The most common MHs were visual illusion (48.4%), which included object misidentification (19.7%), pareidolias (13.9%), and kineptosia (14.8%). Passage hallucinations and presence hallucinations were identified thirty-five (28.7%) and twenty-eight (23.0%) times, respectively.

MHs were described from three aspects: external factors, temporal factors, and contents. The three different types of MHs were consistent in external factors: they could appear at any time, lighting condition, and environment but are more likely to appear indoor during the day when the light is dim (more than 50%). In terms of temporal factors, MHs often persisted for seconds, with a mode of sudden onset and quick disappearance (more than 70%). The frequency of MHs ranged from daily to less than once a week. Most MHs happened 1 ~ 6 times per week; however, 41.7% of object misidentification happened every day. Until they were included in this study, the patients experienced almost all types of MHs for more than 1 month, and some even existed for many years. Compared with the disease duration of PD, 22.1% of MHs started before the onset of the first Parkinsonian motor symptoms. In the case of content, many of the MHs were somewhat blurred (more than 76.4%) and were normal in size (more than 88.5%). Presence hallucinations were described to be a feeling that an unfamiliar person (78.6%) or a familiar lived person (14.3%) is standing behind, often when the patient is focusing on a task. These “people” can be different or wearing different clothes each time, but the colors are mostly monotonous (85.7%). Among the subjects with passage hallucinations, almost all experienced a fleeting image passing that is recognized as an unfamiliar person (51.4%), an animal (17.1%), or an object (8.6%). This kind of flashing image is often stereotyped and without specific color, thus causing the patients to look back. Occasionally because of the flashing too fast, some patients cannot even describe the image (17.1%). Object misidentifications referred to misidentifying an object as a person because of certain similarities in form. The “person” is also stereotyped and without specific color. Pareidolias involved either unfamiliar people (64.7%) or animals (29.4%), and patients usually report seeing a human face or a small animal in a patterned bed sheet or a painting. Kineptosias refer to stationary objects seen in motion, and 66.7% of these illusions have the same color as the original object.

### 3.2. Comparison between PD-MH and PD-NH Groups

Table 2 shows the comparison of demographic information and motor symptom score for PD-MH and PD-NH groups. Among the 215 patients, 74 (39 males (52.7%) and 35 (47.3%) females) had isolated MHs (PD-MH), and 141 patients (68 males (48.2%) and 73 (51.8%) females) did not experience any hallucinations (PD-NH). These patients were corresponded in age, gender, percentage of living alone, education level, BMI, allergies, and personal living habits (e.g., history of smoking; drinking alcohol, coffee, or tea; and daily exercise). Differences in chronic medical history such as hypertension, diabetes, and lacunar infarction were not significant. No substantial discrepancies were noted in terms of family history of PD, predominance of motor symptoms, age of onset, H–Y stage, and UPDRS III on and off scores. The PD-MH group had longer disease duration than the PD-NH group (*P* = 0.011). In terms of PD treatment, LEDD was significantly different between the two groups (P = 0.038). The PD-MH group had higher percentage of levodopa and dopamine-receptor agonist use than the PD-NH group (*P* = 0.013, 0.042), while the use of monoamine oxidase-B (MAO-B) inhibitors, catechol-O-methyltransferase (COMT) inhibitors, amantadine, and benzhexol did not differ between the two.

The nonmotor symptoms of PD-MH and PD-NH groups are summarized in Table 3. Prior to the control for the effect of disease duration and LEDD, the patients with isolated MHs suffered from a great burden of other nonmotor symptoms (*P* < 0.0001). PDSS and RBDSQ analysis indicated that the PD-MH group had more severe sleep problems, especially RBD, than the PD-NH group (*P* < 0.001, *P* = 0.001). The scores of HAMA were relatively high in the minor hallucinators (*P* = 0.034); however, no significant differences in total HAMD scores, total MoCA scores, and MoCA subdomain scores were observed between the groups. The differences in HAMA (*P* = 0.058) scores disappeared after controlling for the effect of disease duration and LEDD.

### 3.3. Risk Factors of MHs

Logistic regression analysis in Table 4 shows that the higher score in RBDSQ (odds ratio (OR) 1.136, 95% confidence interval (CI) 1.018–1.268) was significantly associated with a great risk of MHs (Table 4). Higher scores in PDQ39 (OR 1.014, 95% CI 1.000–1.029) may be a risk factor of MHs. A good performance in PDSS (OR 0.988, 95% CI 0.976-0.999) may protect against MHs. In addition, disease duration, use of levodopa and dopamine agonists, cognitive performance, and depression symptoms were not closely related to MHs.

## 4. Discussion

PDP is a continuum progression that begins with MHs, then evolves to major hallucinations with insight, gradual insight loss, and eventually turns to delusions. The chronological sequence of psychotic symptom is consistent with the Braak pathology of Lewy bodies from the brainstem to the forebrain systems [28]. Although MHs and well-structured VHs have timing consistency, whether they share the same underlying mechanism is still being debated. In this study, the psychosis spectrum was completely observed, and the cooccurrence of two or more psychotic symptoms was not rare. This finding proved from the side that the two hallucinations may have the same mechanism. Consistent with previous findings [4, 6], MHs were the most common psychotic symptom. This phenomenon has never been spontaneously reported in the present study. After a face-to-face interview, MHs were detected in 38.9% of the patients with PD. The previously reported prevalence of MHs ranged from 5.1% to 42% [6, 16]. This wide prevalence variance is predominantly due to the use of different research methods and objects. MH is not a newly found phenomenon in PD but is underestimated because studies on PDP mainly focused on well-structured VHs [6]. The use of different diagnostic criteria and rating scales led to varying results in MH research. Some works did not include all the three types of MH phenomena under the term of “minor hallucination” because of their different diagnostic criteria [29, 30]. Meanwhile, rating scales play a remarkable role in comprehensively identifying and describing MHs. MH is actually a subjective feeling. If not for the doctor's inquiry, then only a few patients would take the initiative to report the presence of MHs [16] either because it did not cause any discomfort or because the patient is afraid of being considered insane. In view of this definition, present results are reliable because all the patients with PD were screened first and then asked in detail about the characteristics of MHs.

Visual illusion was the most common type of MHs in patients with PD, followed by passage hallucinations and presence hallucinations. In particular, object misidentification was the most common type of visual illusion, followed by kineptosia and pareidolia. Two or more types of MHs occurring as comorbidity are highly common. These minor phenomena are typically described to be blurred, stereotyped, and normal-sized. Presence hallucinations are likely to have unfamiliar people standing nearby, and passage hallucinations have animals or objects passing by. Object misidentifications were described as misidentifying an object as an unfamiliar person or another object. Pareidolias were reported as seeing a specific human face or an animal from a complex pattern. Kineptosia mainly involves objects. Presence hallucinations and passage hallucinations are more likely to be in black and white, whereas visual illusions can be polychromatic. These three MH phenomena generally varied in contents but usually occur indoors when the light is dim during the day. These events often appear suddenly, last a few seconds, and occur more than once a week.These phenomena began more than 1 month ago and some even began before the onset of motor symptoms. The MHs presented in the premotor phase have advanced the spectrum of PDP, which was thought to only appear in advanced stage of PD [4, 31, 32]. Therefore, MHs may be a potential marker to differentiate PD from other non-Lewy-body causes of parkinsonism years predate the onset of Parkinsonian motor symptoms [16].

In this study, the patients with MHs showed differences in disease duration and LEDD, especially levodopa and dopamine agonists, which are not independent predictors of MHs. This result was consistent with previous findings [2, 30]. Well-structured VH was associated with long disease duration rather than the age at onset [33]. By contrast, disease duration was not an important predictor of MHs. MHs that appear in the premotor phase can validate this view [16]. Evidence has emerged that PD medications are only a predisposing factor in hallucinations but exhibit no decisive roles. Ravina et al. [4] concluded in a review that the simultaneous occurrence of PD medication and psychosis does not necessarily imply a relationship of causality. The interaction between disease-related factors and medications can explain the characteristics of psychosis. Nonetheless, reasonably reduction in antiparkinsonism medications is recommended when the hallucination becomes distressing. After the adjustment for medications and disease duration, the MH group showed greater nonmotor symptom burden and poorer life quality than the NH group. Therefore, the patients with MHs were likely to suffer from sleep disorders, especially RBD, and tend to have more anxious states rather than depression.

Numerous articles have proved that RBD and MHs are both relevant, and RBD is a predictor of MHs [2, 15, 16, 32]. Morgante et al. [34] conducted a 2-year follow-up study and found that the frequency of RBD gradually increased in patients with PDP. Forsaa et al. [32] reported that PD patients with RBD at baseline are three times more likely to develop psychotic symptoms after a 12-year follow-up. RBD is attributed to the dysfunction of the subcoeruleus nucleus in the mesopontine tegmentum and the reticular formation of the ventral medial medulla [35]. Given that RBD and psychosis are predictors of Lewy body pathology, the association of hallucination with RBD supports the idea that brainstem Lewy body pathology is associated with MHs in PD [2]. Gallagher et al. [36] proposed that the occurrence of VHs may due to the intrusion of REM dream imagery into wakefulness and emergence of internally generated imagery under the circumstance of darkness and a low level of wakefulness just after arousal from sleep [36, 37]. This finding explains why minor phenomena usually emerge under dimly lit conditions.

PDSS was also found to be closely associated with MHs in this population. Compared with the nonhallucinators, the minor hallucinators suffered more from sleep disturbance, a dependent risk factor of MHs. Pappert et al. [38] also found that more than 80% of patients with hallucinations have complaint in their sleep quality. Contrary to previous findings [39], the current study found a correlation between sleep disturbance and MHs. A possible explanation is that previous studies did not include MHs as a kind of hallucinations. Hence, the mechanism remains unclear. Sleep disturbance and hallucinations are unlikely to belong to a continuum [3, 38]. Given the relationship between sleep disorders and MHs, the impaired brainstem regulation of the sleep–wake cycle with fluctuating vigilance has been proposed as an explanation for the generation of MHs [36]. In the current study, PDQ39 tends to be an independent risk factor of MHs, indicating an interaction between life quality and MHs. Kulick et al. [7] found that the life quality of patients with MHs is as poor as those with well-structured VHs. This remind clinicians to pay attention to the life quality of the patients, although health-related life quality has not been routinely associated with MHs in incidence and prevalence studies. Large sample size studies are needed to verify this novel discovery.

The relationship between cognitive impairment and well-structured VHs has been verified [27, 28], whereas the relationship between MHs and cognition remains controversial. Llebaria et al. [40] found that patients with major VHs with insight perform poorly in action verbal fluency task, and this feature is attributed to predominant frontal striatal impairment. Meanwhile, patients with major VHs without insight performed poorly in the PD-Cognitive Rating Scale posterior cortical score and the clock copying item, and this characteristic is ascribed to the impairment of posterior cortical areas. Nevertheless, no difference was observed between minor hallucinators and nonhallucinators. In their 2-year longitudinal neuropsychological study, Morgante et al. [34] found no difference in cognitive function in early stage between hallucinators and nonhallucinators; however, the cognition of patients with hallucinations gradually declined over time. Omoto et al. [37] reported that cognitive impairment is associated with MHs appearing at daytime but not those appearing at nighttime. Given that their study included 100 patients, a large population research is needed to prove this finding. In the present cohort, no difference in global cognitive function or any cognitive domain was found between patients with MHs and those without any hallucination. The simple cognition deficient is not sufficient to explain the occurrence of MHs. However, RBD is related to MH and cognition; therefore, MHs should be considered as a potential indicator of dementia.

MHs were not correlated with depressive symptoms in our population, and this result is consistent with previous findings [15, 16, 37, 41, 42]. By contrast, a cross-sectional analysis based on 199 patients with PD found that the MH group got higher score in depression test than the NH group [7]. Considering that depression is a risk factor of well-structured VHs, additional longitudinal studies are needed to focus on the changes.

As a relatively subtly symptom in PD, MH has always been clinically ignored. Minor phenomena are intermittent but are likely to recur and continue [4]. MHs may be the tip of the iceberg which have major clinical implications [6] and signal a more malignant disease course including progressive psychiatric symptoms and eventually dementia. Given that this phenomenon can occur in the premotor phase, identifying patients with MHs as an at-risk population and establishing the prognostic implications of MHs in PD are necessary [7].

This study has some limitations. First, rather than cross-section research, a longitudinal study is suitable for observing the natural course of MHs because this phenomenon gradually progresses to severe psychotic symptoms. A long-term follow-up will be conducted for this cohort to observe their psychotic state and the effects of disease duration, PD medications, emotions, and RBD on the outcome. Second, the relatively small sample number of 262 may narrow the strength of the identified relationships that were evidenced in the study. Third, a unified assessment scale must be established to fully and comprehensively evaluate the clinical characteristics of MHs.

## 5. Conclusion

A high prevalence of MHs was observed in patients with PD. Patients with MHs have greater burden in a range of nonmotor symptoms and life quality than those without hallucinations. This study provides evidence that MHs are mainly associated with RBD, sleep quality, and health-related life quality. Whether MHs are related to disease duration, PD medication, cognitive impairment, and depression must be further investigated. Future studies focused on MHs, which was overlooked before, need to confirm and expand the related clinical factors, which will offer strategies to improve the life quality of patients with PD.

## Figures and Tables

**Table 1:** Characteristics and contents of minor hallucination.

	Presence hallucination	Passage hallucination	Visual illusion			
			Object misidentification	Pareidolia	Kineptosia	Sum
Number	28 (23.0%)	35 (28.7%)	24 (19.7%)	17 (13.9%)	18 (14.8%)	59 (48.4%)
Appearance time						
Daytime	15 (53.6%)	24 (68.6%)	11 (45.8%)	11 (64.7%)	8 (44.4%)	30 (50.8%)
Nighttime	7 (25.0%)	4 (11.4%)	8 (33.3%)	2 (11.8%)	8 (44.4%)	18 (30.5%)
Both	6 (21.4%)	7 (20.0%)	5 (20.8%)	4 (23.5%)	2 (11.1%)	11 (18.6%)
Lighting						
Bright	7 (25.0%)	12 (34.3%)	6 (25.0%)	8 (47.1%)	7 (38.9%)	21 (35.6%)
Dim	18 (64.3%)	18 (51.4%)	17 (70.8%)	8 (47.1%)	9 (50.0%)	34 (57.6%)
Dark	2 (7.1%)	4 (11.4%)	0 (0.0%)	1 (5.9%)	2 (11.1%)	3 (5.1%)
Variable	1 (3.6%)	1 (2.9%)	1 (4.2%)	0 (0.0%)	0 (0.0%)	1 (1.7%)
Environment						
Indoor	24 (85.7%)	24 (68.6%)	18 (75.0%)	16 (94.1%)	16 (88.9%)	50 (84.7%)
Outdoor	1 (3.6%)	9 (25.7%)	4 (16.7%)	1 (5.9%)	0 (0.0%)	5 (8.5%)
Variable	3 (10.7%)	2 (5.7%)	2 (8.3%)	0 (0.0%)	2 (11.1%)	4 (6.8%)
Onset						
Sudden	23 (82.1%)	33 (94.3%)	24 (100.0%)	14 (82.4%)	18 (100.0%)	56 (94.9%)
Gradual	5 (17.9%)	2 (5.7%)	0 (0.0%)	3 (17.6%)	0 (0.0%)	3 (5.1%)
Lasting time						
Seconds	23 (82.1%)	35 (100.0%)	17 (70.8%)	13 (76.5%)	15 (83.3%)	45 (76.3%)
Minutes	5 (17.9%)	0 (0.0%)	7 (29.2%)	4 (23.5%)	3 (16.7%)	14 (23.7%)
Frequency						
Daily	5 (17.9%)	4 (11.4%)	10 (41.7%)	6 (35.3%)	4 (22.2%)	20 (33.9%)
1–6 times/week	20 (71.4%)	19 (54.3%)	8 (33.3%)	6 (35.3%)	9 (50.0%)	23 (39.0%)
<1/week	3 (10.7%)	12 (34.3%)	6 (25.0%)	5 (29.4%)	5 (27.8%)	16 (27.1%)
Duration						
<1 month	0 (0.0%)	0 (0.0%)	0 (0.0%)	1 (5.9%)	0 (0.0%)	1 (1.7%)
1 month-1 year	15 (53.6%)	19 (54.3%)	11 (45.8%)	9 (52.9%)	11 (61.1%)	31 (52.5%)
>1 year	13 (46.4%)	16 (45.7%)	13 (54.2%)	7 (41.2%)	7 (38.9%)	27 (45.8%)
Premotor						
Yes	3 (10.7%)	10 (28.6%)	7 (29.2%)	3 (17.6%)	4 (22.2%)	14 (23.7%)
No	25 (89.3%)	25 (71.4%)	17 (70.8%)	14 (82.4%)	14 (77.8%)	45 (76.3%)
Clarity						
Sharp	1 (3.6%)	4 (11.4%)	3 (12.5%)	3 (17.6%)	4 (22.2%)	10 (16.9%)
Blurry	26 (92.9%)	31 (88.6%)	21 (87.5%)	13 (76.5%)	14 (77.8%)	48 (81.4%)
Variable	1 (3.6%)	0 (0.0%)	0 (0.0%)	1 (5.9%)	0 (0.0%)	1 (1.7%)
Size						
Normal	26 (92.9%)	31 (88.6%)	23 (95.8%)	16 (94.1%)	18 (100.0%)	57 (96.6%)
Miniaturized	2 (7.1%)	3 (8.6%)	0 (0.0%)	1 (5.9%)	0 (0.0%)	1 (1.7%)
Magnified	0 (0.0%)	0 (0.0%)	1 (4.2%)	0 (0.0%)	0 (0.0%)	1 (1.7%)
Variable	0 (0.0%)	1 (2.9%)	0 (0.0%)	0 (0.0%)	0 (0.0%)	0 (0.0%)
Stereotyped						
Yes	12 (42.9%)	23 (65.7%)	14 (58.3%)	10 (58.8%)	11 (61.1%)	35 (59.3%)
No	13 (46.4%)	10 (28.6%)	9 (37.5%)	7 (41.2%)	6 (33.3%)	22 (37.3%)
Cannot describe	3 (10.7%)	2 (5.7%)	1 (4.2%)	0 (0.0%)	1 (5.6%)	2 (3.4%)
Color						
Black and white	24 (85.7%)	30 (85.7%)	15 (62.5%)	11 (64.7%)	6 (33.3%)	32 (54.2%)
Multiple colors	4 (14.3%)	5 (14.3%)	9 (37.5%)	6 (35.3%)	12 (66.7%)	27 (45.8%)
Content						
Familiar lived people	4 (14.3%)	1 (2.9%)	0 (0.0%)	0 (0.0%)	0 (0.0%)	0 (0.0%)
Unfamiliar people	22 (78.6%)	18 (51.4%)	12 (50.0%)	11 (64.7%)	0 (0.0%)	23 (39.0%)
Deceased relatives	0 (0.0%)	1 (2.9%)	0 (0.0%)	0 (0.0%)	0 (0.0%)	0 (0.0%)
Animals	0 (0.0%)	6 (17.1%)	3 (12.5%)	5 (29.4%)	2 (11.1%)	10 (16.9%)
Objects	1 (3.6%)	3 (8.6%)	8 (33.3%)	1 (5.9%)	16 (88.9%)	25 (42.4%)
Cannot describe	1 (3.6%)	6 (17.1%)	1 (4.2%)	0 (0.0%)	0 (0.0%)	1 (1.7%)

**Table 2:** Demographic parameters in the PD-MH and PD-NH patients.

	PD-total	PD-MH	PD-NH	Value	*P*
Number, *n* (%)	215 (100%)	74 (34.4%)	141 (65.6%)		
Age, y, mean ± SD	66.17 ± 9.43	65.70 ± 8.07	66.42 ± 10.09	4931	0.509^a^
Gender, *n* (%)					
Male	107 (49.8%)	39 (52.7%)	68 (48.2%)	0.389	0.533^b^
Female	108 (50.2%)	35 (47.3%)	73 (51.8%)		
Percentage of live alone, *n* (%)	21 (9.8%)	4 (5.4%)	17 (12.1%)	2.436	0.119^b^
Education, *n* (%)					
Illiterate	31 (14.4%)	8 (10.8%)	23 (16.3%)	5202.5	0.971^b^
Primary school	31 (14.4%)	12 (16.2%)	19 (13.5%)		
Middle school	108 (50.2%)	41 (55.4%)	67 (47.5%)		
College or above	45 (20.9%)	13 (17.6%)	32 (22.7%)		
BMI, mean ± SD	23.71 ± 3.51	23.55 ± 3.52	23.79 ± 3.51	-0.471	0.638^c^
Allergies, *n* (%)	32 (14.9%)	9 (12.2%)	23 (16.3%)	0.66	0.417^b^
Smoker, *n* (%)	59 (27.4%)	21 (28.4%)	38 (27.0%)	0.05	0.824^b^
Alcohol intake, *n* (%)	28 (13.0%)	11 (14.9%)	17 (12.1%)	0.338	0.561^b^
Drinking tea, *n* (%)	41 (19.1%)	15 (20.3%)	26 (18.4%)	0.105	0.745^b^
Drinking coffee, *n* (%)	14 (6.5%)	7 (9.5%)	7 (5.0%)	1.603	0.247^b^
Daily exercise, *n* (%)	111 (51.6%)	37 (50.0%)	74 (52.5%)	0.12	0.729^b^
Hypertension, *n* (%)	80 (37.2%)	26 (35.1%)	54 (38.3%)	0.208	0.649^b^
Diabetes, *n* (%)	31 (14.4%)	11 (14.9%)	20 (14.2%)	0.018	0.893^b^
Lacunar infarction, *n* (%)	81 (37.7%)	26 (35.1%)	55 (39.0%)	0.31	0.578^b^
Family history of PD, *n* (%)	23 (10.7%)	7 (9.5%)	16 (11.3%)	0.181	0.67^b^
Predominance of motor symptoms, *n* (%)					
None	19 (8.8%)	7 (9.5%)	12 (8.5%)	1.179	0.555^b^
Left	101 (47.0%)	31 (41.9%)	70 (49.6%)		
Right	95 (44.2%)	36 (48.6%)	59 (41.8%)		
Age of onset, y, mean ± SD	60.88 ± 10.84	59.64 ± 9.47	61.54 ± 11.47	4548	0.122^a^
Disease duration, y, mean ± SD	5.35 ± 4.48	6.14 ± 4.26	4.93 ± 4.56	4121	*0.011* ^a^
PD treatment					
Levodopa, *n* (%)	158 (73.5%)	62 (83.8%)	96 (68.1%)	6.139	*0.013* ^b^
Dopamine agonists, *n* (%)	122 (56.7%)	49 (66.2%)	73 (51.8%)	4.125	*0.042* ^b^
MAO-B inhibitors, *n* (%)	29 (13.5%)	12 (16.2%)	17 (12.1%)	0.72	0.396^b^
COMT inhibitors, *n* (%)	32 (14.9%)	14 (18.9%)	18 (12.8%)	1.45	0.228^b^
Amantadine, *n* (%)	40 (18.6%)	17 (23.0%)	23 (16.3%)	1.422	0.233^b^
Benzhexol, *n* (%)	15 (7.0%)	6 (8.1%)	9 (6.4%)	0.223	0.637^b^
LEDD, mg, mean ± SD	441.91 ± 389.02	492.04 ± 330.26	415.60 ± 415.25	4323	*0.038* ^a^
H-Y stage, mean ± SD	2.41 ± 0.61	2.52 ± 0.60	2.34 ± 0.61	4447	0.063^a^
UPDRS III on, mean ± SD	19 ± 9.65	18.93 ± 9.52	19.04 ± 9.75	5149.5	0.876^a^
UPDRS III off, mean ± SD	29.19 ± 13.05	31.11 ± 14.92	28.18 ± 11.88	4646	0.187^a^

^a^Mann-Whitney *U* test. ^b^Chi-square test. ^c^Student's *t*-test. ^d^Significant results are highlighted in italic (*P* < 0.05). SD: standard deviation; PD: Parkinson's disease; MH: minor hallucination; NH: no hallucinations; BMI: body mass index; LEDD: levodopa equivalent daily dose; H–Y stage: Hoehn and Yahr stage; UPDRS III: the Unified Parkinson's Disease Rating Scale part III.

**Table 3:** Clinical characteristics of the PD-MH and PD-NH patients.

	PD-total	PD-MH	PD-NH	Value	*P*	Adjusted value^b^	Adjusted *P*^b^
NMS-Quest, mean ± SD	11.83 ± 5.13	14.16 ± 5.22	10.61 ± 4.65	3157	*<0.0001* ^a^	23.154	*<0.0001* ^a^
PDSS, mean ± SD	107.73 ± 28.59	99.52 ± 28.64	112.04 ± 27.70	3691.5	*<0.001* ^a^	7.486	*0.007* ^a^
MoCA, mean ± SD	24.04 ± 5.22	24.47 ± 4.58	23.82 ± 5.53	5080.5	0.752^a^	0.675	0.412^a^
Visuospatial/executive, mean ± SD	2.96 ± 1.71	3.11 ± 1.58	2.88 ± 1.77	4915	0.478^a^	0.776	0.379^a^
Naming, mean ± SD	2.76 ± 0.62	2.82 ± 0.53	2.72 ± 0.66	4836.5	0.166^a^	1.028	0.312^a^
Attention, mean ± SD	5.43 ± 1.12	5.5 ± 0.95	5.39 ± 1.20	5095.5	0.724^a^	0.347	0.556^a^
Language, mean ± SD	2.69 ± 0.66	2.77 ± 0.51	2.65 ± 0.73	4948	0.389^a^	1.241	0.267^a^
Abstraction, mean ± SD	1.45 ± 0.75	1.51 ± 0.71	1.42 ± 0.78	4930.5	0.447^a^	0.773	0.380^a^
Delayed memory, mean ± SD	3.12 ± 1.51	3.09 ± 1.47	3.13 ± 1.53	5061.5	0.714^a^	0.007	0.931^a^
Orientation, mean ± SD	5.64 ± 0.83	5.66 ± 0.75	5.62 ± 0.88	5119.5	0.751^a^	0.084	0.773^a^
HAMA, mean ± SD	6.91 ± 4.65	7.81 ± 4.65	6.44 ± 4.59	4302.5	*0.034* ^a^	3.624	0.058^a^
HAMD, mean ± SD	7.98 ± 4.88	8.30 ± 4.88	7.82 ± 4.89	4961	0.554^a^	0.180	0.672^a^
PDQ39, mean ± SD	44.84 ± 26.89	52.20 ± 29.38	40.97 ± 24.73	4074.5	*0.008* ^a^	5.485	*0.020* ^a^
RBDSQ, mean ± SD	3.26 ± 2.79	4.12 ± 2.80	2.81 ± 2.68	3741.5	*0.001* ^a^	9.114	*0.003* ^a^

^a^Mann-Whitney *U* test. ^b^The value and *P* are calculated adjusted for disease duration and LEDD. ^c^Significant results are highlighted in italic (*P* < 0.05). SD: standard deviation; PD: Parkinson's disease; MH: minor hallucination; NH: no hallucinations; NMS-Quest: Non-Motor Symptoms Questionnaire; PDSS: the PD Sleep Scale; MOCA: Montreal Cognitive Assessment; HAMA: Hamilton Anxiety Rating Scale; HAMD: Hamilton Depression Rating Scale; PDQ39: Parkinson's Disease Questionnaire-39; RBDSQ: the REM Sleep Behavior Disorder Sleep Questionnaire.

**Table 4:** Logistic regression analyses for independent predictors of minor hallucinations.

	OR	95% CI	*P*
Disease duration	0.984	0.913-1.061	0.672
Levodopa	0.569	0.234-1.382	0.213
Dopamine agonists	0.801	0.386-1.664	0.552
PDSS	0.988	0.976-0.999	*0.032*
MoCA	1.043	0.978-1.112	0.201
HAMD	0.952	0.885-1.024	0.189
RBDSQ	1.136	1.018-1.268	*0.023*
PDQ39	1.014	1.000-1.029	*0.048*

^a^ Significant results are highlighted in italic (*P* < 0.05). OR: odds ratio; CI: confidence interval; PDSS: the PD Sleep Scale; MOCA: Montreal Cognitive Assessment; HAMD: Hamilton Depression Rating Scale; RBDSQ: the REM Sleep Behavior Disorder Sleep Questionnaire; PDQ39: Parkinson's Disease Questionnaire-39.

## Data Availability

The data supporting the findings of this study are included in the article; further inquiries can be directed to the corresponding author.

## References

[1] Bloem B. R., Okun M. S., Klein C. (2021). Parkinson's disease. *The Lancet.*.

[2] Barrett M. J., Smolkin M. E., Flanigan J. L., Shah B. B., Harrison M. B., Sperling S. A. (2017). Characteristics, correlates, and assessment of psychosis in Parkinson disease without dementia. *Parkinsonism & Related Disorders*.

[3] Fénelon G., Mahieux F., Huon R., Ziégler M. (2000). Hallucinations in Parkinson’s disease: prevalence, phenomenology and risk factors. *Brain*.

[4] Ravina B., Marder K., Fernandez H. H. (2007). Diagnostic criteria for psychosis in Parkinson’s disease: report of an NINDS, NIMH work group. *Movement Disorders*.

[5] Nishio Y., Yokoi K., Hirayama K. (2018). Defining visual illusions in Parkinson's disease: Kinetopsia and object misidentification illusions. *Parkinsonism & Related Disorders*.

[6] Lenka A., Pagonabarraga J., Pal P. K., Bejr-Kasem H., Kulisvesky J. (2019). Minor hallucinations in Parkinson disease: a subtle symptom with major clinical implications. *Neurology*.

[7] Kulick C. V., Montgomery K. M., Nirenberg M. J. (2018). Comprehensive identification of delusions and olfactory, tactile, gustatory, and minor hallucinations in Parkinson's disease psychosis. *Parkinsonism & Related Disorders*.

[8] Williams D. R., Lees A. J. (2005). Visual hallucinations in the diagnosis of idiopathic Parkinson's disease: a retrospective autopsy study. *Lancet Neurology*.

[9] O'Brien J., Taylor J. P., Ballard C. (2020). Visual hallucinations in neurological and ophthalmological disease: pathophysiology and management. *Journal of Neurology, Neurosurgery, and Psychiatry*.

[10] Gibson G., Mottram P. G., Burn D. J. (2013). Frequency, prevalence, incidence and risk factors associated with visual hallucinations in a sample of patients with Parkinson’s disease: a longitudinal 4-year study. *International Journal of Geriatric Psychiatry*.

[11] Goetz C. G., Stebbins G. T. (1995). Mortality and hallucinations in nursing home patients with advanced Parkinson’s disease. *Neurology*.

[12] Mosimann U. P., Rowan E. N., Partington C. E. (2006). Characteristics of visual hallucinations in Parkinson disease dementia and dementia with Lewy bodies. *The American Journal of Geriatric Psychiatry*.

[13] Goetz C. G., Fan W., Leurgans S., Bernard B., Stebbins G. T. (2006). The malignant course of “benign hallucinations” in Parkinson disease. *Archives of Neurology*.

[14] Collerton D., Perry E., McKeith I. (2005). Why people see things that are not there: a novel perception and attention deficit model for recurrent complex visual hallucinations. *The Behavioral and Brain Sciences*.

[15] Bejr-kasem H., Pagonabarraga J., Martínez-Horta S. (2019). Disruption of the default mode network and its intrinsic functional connectivity underlies minor hallucinations in Parkinson’s disease. *Movement Disorders*.

[16] Pagonabarraga J., Martinez-Horta S., Fernández de Bobadilla R. (2016). Minor hallucinations occur in drug-naive Parkinson’s disease patients, even from the premotor phase. *Movement Disorders*.

[17] Hughes A., Daniel S., Kilford L., Lees A. (1992). Accuracy of clinical diagnosis of idiopathic Parkinson’s disease: a clinico-pathological study of 100 cases. *Journal of Neurology, Neurosurgery, and Psychiatry*.

[18] Arnaoutoglou N. A., O’Brien J. T., Underwood B. R. (2019). Dementia with Lewy bodies -- from scientific knowledge to clinical insights. *Nature Reviews. Neurology*.

[19] Goetz C. G., Tilley B. C., Shaftman S. R. (2008). Movement Disorder Society-sponsored revision of the Unified Parkinson’s Disease Rating Scale (MDS-UPDRS): scale presentation and clinimetric testing results. *Movement Disorders*.

[20] Tomlinson C. L., Stowe R., Patel S., Rick C., Gray R., Clarke C. E. (2010). Systematic review of levodopa dose equivalency reporting in Parkinson’s disease. *Movement Disorders*.

[21] Wu Z., Jiang X., Zhong M. (2020). Wearable sensors measure ankle joint changes of patients with Parkinson’s disease before and after acute levodopa challenge. *Parkinsons Disease*.

[22] Chaudhuri K. R., Martinez-Martin P., Schapira A. H. (2006). International multicenter pilot study of the first comprehensive self-completed nonmotor symptoms questionnaire for Parkinson’s disease: the NMSQuest study. *Movement Disorders*.

[23] Gill D. J., Freshman A., Blender J. A., Ravina B. (2008). The Montreal Cognitive Assessment as a screening tool for cognitive impairment in Parkinson’s disease. *Movement Disorders*.

[24] Chaudhuri K., Pal S., DiMarco A. (2002). The Parkinson’s disease sleep scale: a new instrument for assessing sleep and nocturnal disability in Parkinson’s disease. *Journal of Neurology, Neurosurgery, and Psychiatry*.

[25] Stiasny-Kolster K., Mayer G., Schafer S., Moller J. C., Heinzel-Gutenbrunner M., Oertel W. H. (2007). The REM sleep behavior disorder screening questionnaire--a new diagnostic instrument. *Movement Disorders*.

[26] Peto V., Jenkinson C., Fitzpatrick R., Greenhall R. (1995). The development and validation of a short measure of functioning and well being for individuals with Parkinson’s disease. *Quality of Life Research*.

[27] Zhu J., Shen B., Lu L. (2017). Prevalence and risk factors for visual hallucinations in Chinese patients with Parkinson's disease. *Journal of the Neurological Sciences*.

[28] ffytche D. H., Creese B., Politis M. (2017). The psychosis spectrum in Parkinson disease. *Nature Reviews. Neurology*.

[29] Fénelon G., Soulas T., Zenasni F., LCD L. (2010). The changing face of Parkinson’s disease-associated psychosis: a cross-sectional study based on the new NINDS-NIMH criteria. *Movement Disorders*.

[30] Clegg B. J., Duncan G. W., Khoo T. K. (2018). Categorising visual hallucinations in early Parkinson’s disease. *Journal of Parkinson's Disease*.

[31] Goetz C. G., Leurgans S., Pappert E. J., Raman R., Stemer A. B. (2001). Prospective longitudinal assessment of hallucinations in Parkinson’s disease. *Neurology*.

[32] Forsaa E. B., Larsen J. P., Wentzel-Larsen T. (2010). A 12-year population-based study of psychosis in Parkinson disease. *Archives of Neurology*.

[33] Fenelon G., Alves G. (2010). Epidemiology of psychosis in Parkinson's disease. *Journal of the Neurological Sciences*.

[34] Morgante L., Colosimo C., Antonini A. (2012). Psychosis associated to Parkinson’s disease in the early stages: relevance of cognitive decline and depression. *Journal of Neurology, Neurosurgery, and Psychiatry*.

[35] Iranzo A. (2018). The REM sleep circuit and how its impairment leads to REM sleep behavior disorder. *Cell and Tissue Research*.

[36] Gallagher D. A., Parkkinen L., O'Sullivan S. S. (2011). Testing an aetiological model of visual hallucinations in Parkinson’s disease. *Brain*.

[37] Omoto S., Murakami H., Shiraishi T., Bono K., Umehara T., Iguchi Y. (2021). Risk factors for minor hallucinations in Parkinson’s disease. *Acta Neurologica Scandinavica*.

[38] Pappert E., Goetz C., Niederman F., Raman R., Leurgans S. (1999). Hallucinations, sleep fragmentation, and altered dream phenomena in Parkinson’s disease. *Movement Disorders*.

[39] Goetz C. G., Ouyang B., Negron A., Stebbins G. T. (2010). Hallucinations and sleep disorders in PD: ten-year prospective longitudinal study. *Neurology*.

[40] Llebaria G., Pagonabarraga J., Martínez-Corral M. (2010). Neuropsychological correlates of mild to severe hallucinations in Parkinson’s disease. *Movement Disorders*.

[41] Pacchetti C., Manni R., Zangaglia R. (2005). Relationship between hallucinations, delusions, and rapid eye movement sleep behavior disorder in Parkinson’s disease. *Movement Disorders*.

[42] Pagonabarraga J., Soriano-Mas C., Llebaria G., Lopez-Sola M., Pujol J., Kulisevsky J. (2014). Neural correlates of minor hallucinations in non-demented patients with Parkinson's disease. *Parkinsonism & Related Disorders*.

